# Dorsal Preservation Rhinoplasty in East Asian Patients: A Case Series with 6-Month Follow-Up

**DOI:** 10.1055/a-2867-5301

**Published:** 2026-07-07

**Authors:** Sungwoo Park, Yong-Seon Hwang, Da Woon Lee

**Affiliations:** 1 Department of Plastic Surgery, AVEC Plastic Surgery Clinic, Seoul, South Korea; 2 65371 Department of Plastic and Reconstructive Surgery, Cheonan Hospital, Soonchunhyang University College of Medicine, Cheonan-si, Chungcheongnam-do, South Korea

**Keywords:** rhinoplasty, preservation rhinoplasty, dorsal preservation, East Asian rhinoplasty, structural rhinoplasty, case series

## Abstract

Dorsal preservation rhinoplasty has recently gained popularity for natural dorsal contours with minimal disruption. However, its application to East Asian noses remains limited due to anatomical features such as thicker skin and weaker cartilage compared to Caucasian noses. We retrospectively reviewed five patients who underwent structural preservation rhinoplasty using push-down or let-down techniques. Structural tip grafting was applied in all cases. Outcomes were assessed using clinical photographs, computed tomography (CT) scans, and patient satisfaction surveys during a 3- to 6-month follow-up period. All patients demonstrated improved dorsal profiles without major complications. A mild recurrence of the dorsal hump occurred in one case. CT analysis showed a mean hump reduction of 2.75 mm. Four patients reported high satisfaction: One reported moderate satisfaction due to tip over-projection. Structural preservation rhinoplasty is feasible in Asians when modified with structural support techniques. CT-based findings support the technique's suitability for Asian nasal anatomy.

## Introduction

Preservation rhinoplasty maintains dorsal aesthetic lines while avoiding traditional humpectomy, thereby reducing postoperative irregularities and preserving nasal patency. 1 2 Dorsal preservation techniques have been primarily applied in Caucasian patients, whose nasal anatomy is generally well-suited to such approaches. 1 3

Recently, hybrid approaches combining dorsal preservation with structural rhinoplasty have been proposed to improve support and predictability in anatomically challenging cases. 4 These methods offer greater versatility when adapted for nasal anatomies distinct from the typical Caucasian profile. East Asian nasal anatomy typically includes weaker cartilage and thicker skin than that of Caucasians. 5 6 As a result, dorsal preservation techniques remain underutilized and understudied in East Asians. 5 6 7

Given growing interest in preservation principles for different nasal anatomies, this study evaluates the feasibility of structural preservation rhinoplasty in five Korean patients, focusing on technique modification, aesthetic and functional outcomes, and the potential role of hybrid preservation methods in East Asians.

## Case

This retrospective case series included five Korean patients (three males and two females) who underwent structural preservation rhinoplasty between November 2024 and March 2025. Patients were carefully selected based on specific anatomical indications, including a high dorsal hump or a mild bony asymmetry. All patients were primary rhinoplasty cases. Preoperative evaluation involved standardized clinical photography and computed tomography (CT) to assess the nasal framework and dorsal contour.

### Surgical Technique

A dorsal preservation approach was employed using either the push-down or let-down technique with conservative bony mobilization to preserve the keystone area and dorsal aesthetic line. Asymmetric lateral osteotomies were performed when bony asymmetry was present.

The septum was exposed via an anterior approach, carefully preserving the mucosal lining between the dorsal septum and upper lateral cartilages. Following a subdorsal resection to reduce dorsal height, septal cartilage was harvested while maintaining a conservative 1.5-cm wide L-shaped strut. The cartilage was utilized to construct bilateral septal extension grafts, serving as the primary structural support for tip projection and rotation. Additionally, conchal cartilage was selectively employed as an onlay or dorsal batten graft to enhance tip and definition for aesthetic refinement.

All surgeries were performed by a single surgeon. Postoperative care included one week of external splinting and follow-up at 1, 3, and 6 months. Dorsal hump height was measured on sagittal CT multiplanar reconstruction images, which allow accurate linear measurements by reconstructing axial data into sagittal planes.

Dorsal hump height was defined as the maximum perpendicular distance from a nasion-to-tip reference line to the most projecting point on the dorsum, based on pre- and postoperative CT scans. All measurements were performed by a single observer using a standardized protocol. This study was approved by the institutional review board (IRB Number 2025-07-030), and written informed consent was obtained from all patients.

### Clinical Outcome

Five patients (mean age: 29.2 years) were included, with indications of a high dorsal hump ( *n* = 3) or an asymmetric bony vault ( *n* = 2). Push-down ( *n* = 3) and let-down ( *n* = 2) techniques were selected based on anatomy. Septal extension grafts were applied in all patients, while conchal cartilage grafts were selectively added based on anatomical need ( Table 1 ).

**Table 1:** Summary of patient characteristics, surgical techniques, and outcomes

Case	Sex/Age	Indication	Dorsal technique	Grafts used	Follow-up	Complication	Satisfaction
1	Male/29	High dorsal hump, Asymmetric bony vault	Let-down	Septal extension graft Dorsal batten graft Tip onlay graft	6 months	Mild hump recurrence	High
2	Female/30	High dorsal hump	Push-down	Septal extension graft Tip onlay graft	6 months	None	Moderate (due to over-projected tip)
3	Male/35	Asymmetric bony vault	Push-down	Septal extension graft Tip onlay graft	3 months	None	High
4	Male/28	High dorsal hump, Asymmetric bony vault	Let-down	Septal extension graft Radix graft Tip onlay graft	3 months	None	High
5	Female/23	Asymmetric bony vault	Push-down	Septal extension graft Dorsal batten graft Tip onlay graft	3 months	None	High

Five Korean patients underwent structural preservation rhinoplasty using either the push-down or let-down technique. Septal extension grafts were used in all cases, with auricular cartilage added selectively for contour refinement. No major complications occurred. One patient had a minor hump recurrence, and one reported moderate satisfaction.

The average follow-up period was 4.2 months (range, 3–6 months). No major complications such as dorsal irregularity, saddle nose, or airway compromise were observed. One patient (Case 1) experienced a mild recurrence of the dorsal hump at 6 months but did not require revision.

Patient satisfaction was reported as high in four cases and moderate in one (Case 2), in which the patient perceived over-projection of the nasal tip.

**Fig. 1:**
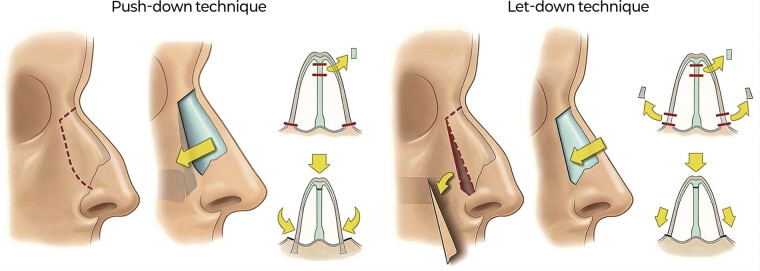
Clinical and radiologic outcomes of Case 1, a 28-year-old male who underwent structural preservation rhinoplasty using the push-down technique. Septal extension graft, dorsal batten graft, and tip onlay graft were applied. Frontal ( **A** ) and lateral views ( **B** ) show a natural dorsal profile and improved tip definition at 6-month follow-up. Sagittal ( **C** ) and axial CT views ( **D** ) confirm dorsal preservation and maintenance of airway patency without irregularity or collapse.

**Fig. 2:**
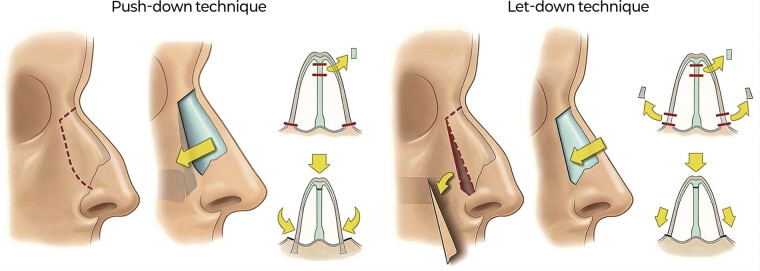
Clinical and radiologic outcomes of Case 4, a 28-year-old male who underwent structural preservation rhinoplasty using the let-down technique. A septal extension graft, radix graft, and tip onlay graft were performed. Postoperative frontal ( **A** ) and lateral views ( **B** ) demonstrate a smoother dorsal line and improved radix-to-tip continuity. Sagittal ( **C** ) and axial CT scans ( **D** ) confirm stable dorsal alignment and maintenance of nasal airway patency at 3-month follow-up.

CT-based dorsal profile analysis was performed in four patients with available postoperative imaging. The mean preoperative hump height was 4.7 mm (range, 2.7–6.2 mm), and the postoperative height was 1.95 mm (range, 1.4–2.2 mm), resulting in an average hump reduction of 2.75 mm. Let-down cases demonstrated a greater reduction (mean 3.8 mm) compared to push-down cases (mean 1.7 mm). These findings supported the preservation of the dorsal aesthetic line with minimal resection and stable postoperative profiles ( Table 2 and Figs. 1 and 2 ). Supplementary Figures S1 to S5 (available in the online version only) display pre- and postoperative sagittal CT images corresponding to each of the five cases. These images illustrate changes in dorsal contour following preservation rhinoplasty. Postoperative imaging was unavailable for Case 3 due to loss to follow-up, and only the preoperative image is shown.

**Table 2:** Computed tomography-based analysis of dorsal hump reduction and surgical technique

Case	Preop hump height (mm)	Postop height (mm)	Hump reduction (mm)	Dorsal technique	Postop CT available
1	6.2	2.1	4.1	Let-down	Yes
2	4.2	1.4	2.8	Push-down	Yes
3	3.3	–	–	Push-down	No
4	5.7	2.2	3.5	Let-down	Yes
5	2.7	2.1	0.6	Push-down	Yes

This table presents preoperative and postoperative computed tomography (CT) measurements of dorsal hump height in four patients. Hump height was defined as the maximum perpendicular distance from the nasion-to-tip line to the most projecting point of the nasal dorsum. The surgical technique and availability of postoperative CT scans are also indicated.

## Discussion

Compared with Caucasian populations, indications for dorsal preservation rhinoplasty in East Asian noses have been limited. 5 6 7 8 In these cases, we evaluated the feasibility of structural preservation rhinoplasty by adapting dorsal preservation principles to individual nasal anatomy.

**Fig. 3:**
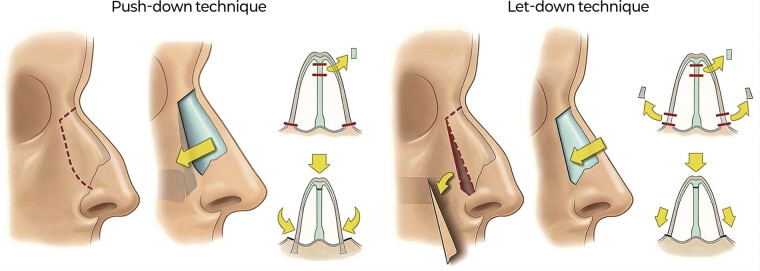
Schematic illustration comparing push-down and let-down techniques in dorsal preservation rhinoplasty. (Left) Push-down technique. Red dashed lines indicate the plan for basal osteotomies to mobilize the entire nasal pyramid. The osseocartilaginous vault (teal area) is depressed downward (yellow arrow) into the piriform aperture without lateral wall resection. The cross-sectional views (inset) show the descent of the intact nasal dorsum. (Right) Let-down technique. A wedge resection of the lateral nasal wall is performed (indicated by the removed dark red segment in the profile view and gray wedges in the cross-section). The nasal dorsum is then lowered into the space created by the resection to reduce the dorsal height.

Preservation rhinoplasty aims to maintain native osteocartilaginous structures. 9 The hump is managed by en bloc mobilization and downward repositioning using the push-down technique, preserving the dorsal aesthetic lines without humpectomy. 1 The push-down technique lowers the dorsum as a single unit, preserving the keystone area, whereas the let-down technique incorporates lateral and transverse osteotomies with wedge resections to allow controlled dorsal descent ( Fig. 3 ). Let-down is generally preferred for larger humps (>4 mm), whereas push-down is suitable for smaller humps. 10 By preserving the keystone and middle vault, these approaches promote smoother dorsal contours and enhance structural stability compared with conventional structural rhinoplasty. 3

Recent studies have also reported favorable aesthetic and functional outcomes with preservation rhinoplasty techniques. 11 12 Hybrid approaches combining dorsal preservation with structural grafting have been proposed to increase stability and adaptability. 4

In contrast to conventional humpectomy, which involves dorsal hump resection, the push-down technique repositions the osseocartilaginous vault downward as a single unit. By preserving the keystone and middle vault integrity, this approach minimizes the risk of complications such as inverted-V deformity, dorsal irregularity, dorsal widening, and midvault collapse. Because it incorporates controlled osteotomies, push-down can simultaneously address dorsal height and lateral bony deviation. These conceptual and technical distinctions allow preservation rhinoplasty to maintain more natural dorsal lines while reducing the need for structural rhinoplasty.

Hybrid approaches integrating preservation of the dorsal aesthetic lines with structural grafting have gained increasing support, particularly in anatomically challenging noses. 4

As emphasized by Toriumi, this concept is particularly relevant in East Asians, in whom pure dorsal preservation may provide insufficient support or projection. By combining the minimal invasiveness of dorsal preservation with the predictability and stability of structural techniques, the hybrid approach allows for both natural contouring and long-term support. Our outcomes are consistent with this concept, demonstrating generally high patient satisfaction with a low incidence of minor complications, such as limited tip over-projection or mild dorsal hump recurrence, none of which required revision. 2 3 13

Although East Asian noses are generally characterized by a low nasal dorsum, thick skin envelope, and weak cartilaginous framework, there exists considerable anatomical variation within this population. For example, Korean patients tend to have thicker skin and a relatively higher radix compared to Southeast Asians, while Japanese patients often exhibit more defined cartilage structure with relatively thin skin. 6

These differences influence the outcomes of preservation rhinoplasty. In patients with moderate hump and strong bony support, push-down techniques may be sufficient, whereas let-down or hybrid approaches may be required in cases with prominent bony hump or weak tip support. Understanding such variation is essential for selecting appropriate techniques and avoiding complications like undercorrection or contour irregularity. 14 Individualized preservation strategies are essential for optimal outcomes in East Asian patients.

In this study, CT-based quantitative analysis confirmed the effectiveness of preservation techniques in dorsal hump management. By using the nasion-to-tip straight line as a baseline, we observed an average hump reduction of 2.75 mm in patients with postoperative imaging. Let-down techniques achieved a greater degree of reduction than push-down, particularly in cases with a more prominent dorsal hump. In one case (Case 5), only 0.6 mm of reduction yielded a satisfactory aesthetic result, emphasizing that even minimal hump adjustments can lead to perceptible aesthetic improvements. These findings suggest that CT can serve as an objective tool to evaluate outcomes and guide technique selection.

However, several limitations should be acknowledged. First, the sample size was small ( *n* = 5), limiting generalizability. Second, the follow-up period was relatively short (3–6 months), which may be insufficient to reliably assess dorsal relapse, tip projection stability, or other delayed complications that can occur after preservation rhinoplasty. As this procedure relies on bony repositioning, relapse may result from gradual shifting during bone healing. Lastly, the retrospective design may have introduced selection bias, as only patients with available imaging and follow-up were included. Further studies with larger cohorts and longer follow-up are necessary to validate these findings and optimize technique selection for East Asians.

In conclusion, this case series supports the feasibility of structural preservation rhinoplasty in selected Korean patients. When combined with appropriate tip support and conservative dorsal manipulation, preservation techniques can achieve reliable aesthetic outcomes without major complications. The incorporation of CT-based objective measurements further enhances the reproducibility and clarity of outcomes in populations with unique anatomical challenges. As hybrid strategies continue to evolve, future studies with larger cohorts and longer follow-up will be essential to refine surgical indications and optimize technique selection for East Asian rhinoplasty.
