# Supplementary material for: Dorsal Preservation Rhinoplasty in East Asian Patients: A Case Series with 6-Month Follow-Up
Source: Arch Plast Surg. 2026 Jul 7;53(4):347–51. doi: 10.1055/a-2867-5301 (PMC13463898; doi:10.1055/a-2867-5301)
Supplement: Supplementary file 1 [file 10-1055-a-2867-5301_29099268.pdf]

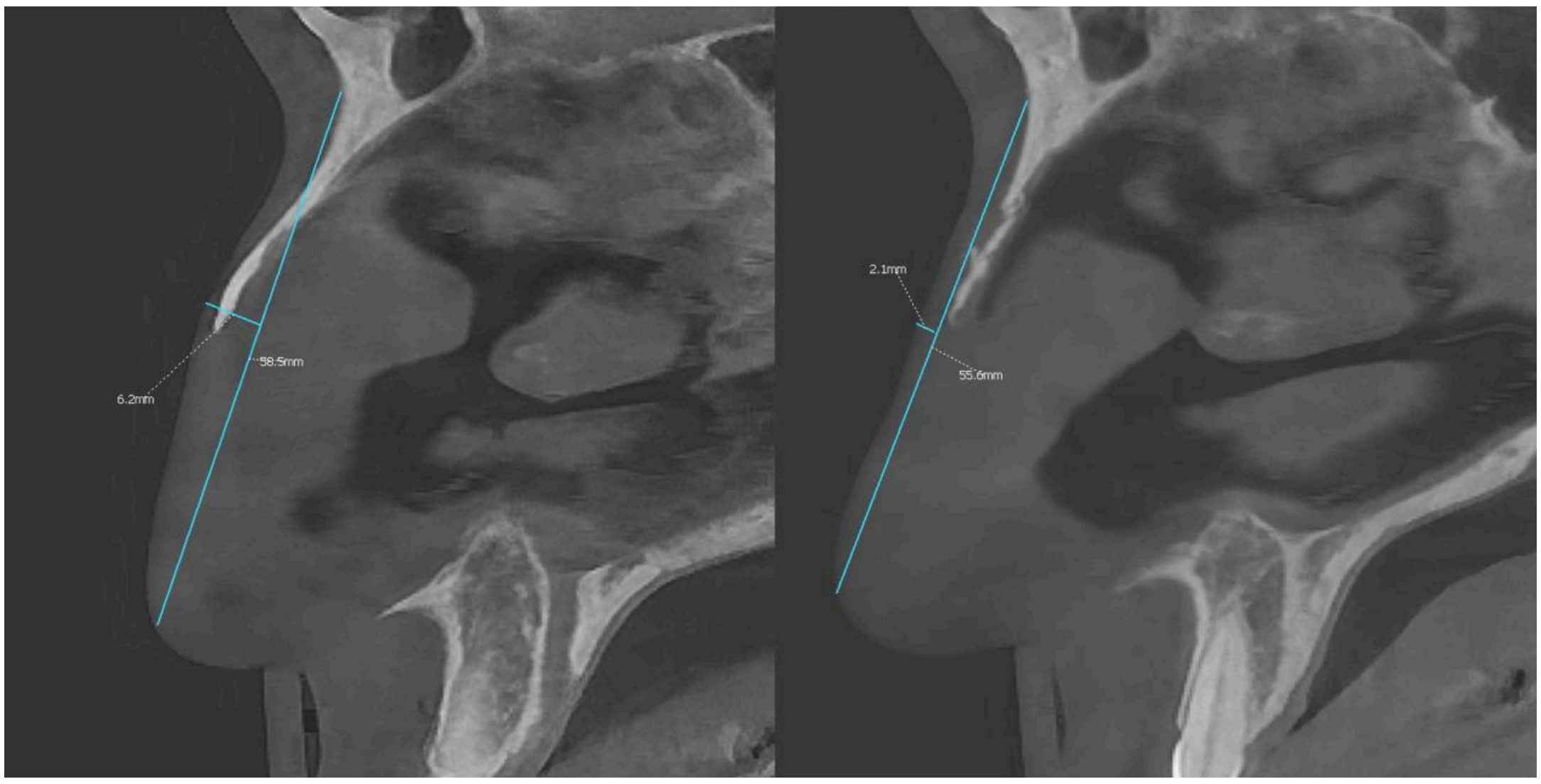

Supplementary Fig. S1. Case 1 – Preoperative and postoperative sagittal CT images showing dorsal hump reduction following let-down technique (follow-up: 6 months).

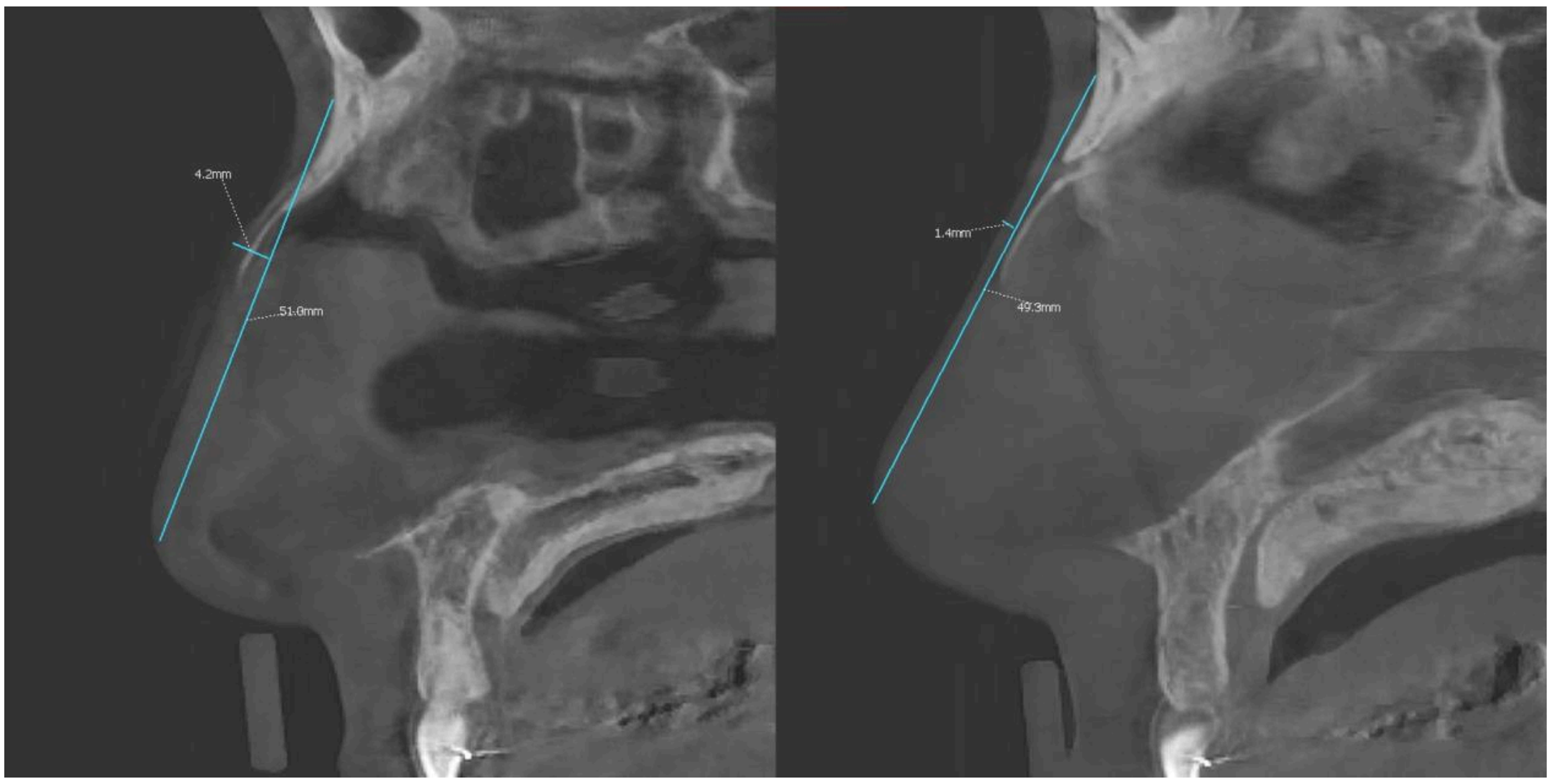

Supplementary Fig. S2. Case 2 – Preoperative and postoperative sagittal CT images after push-down technique (follow-up: 3 months).

Arch Plast Surg | DOI 10.1055/a-2867-5301 | © The Author(s).

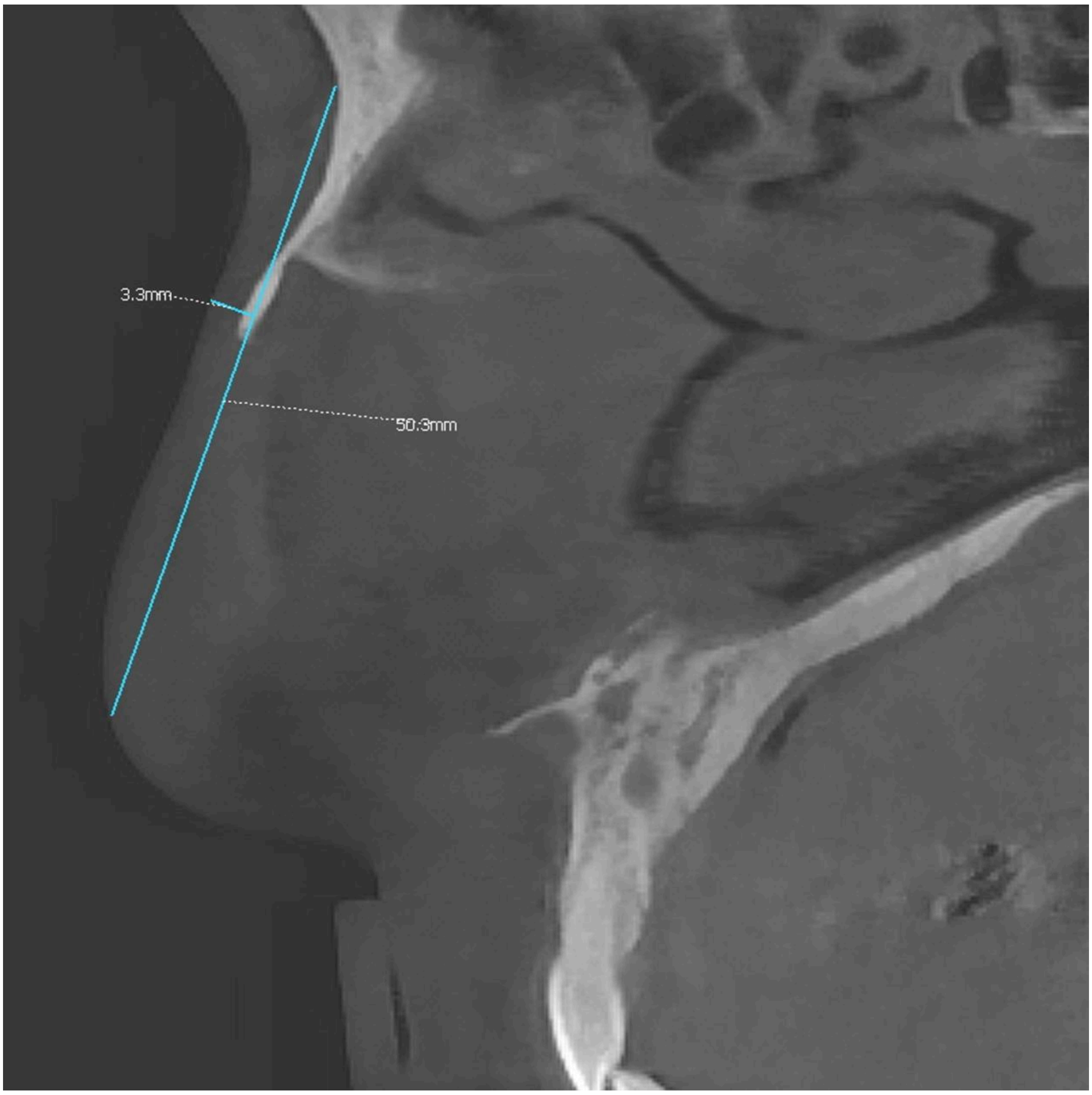

Supplementary Fig. S3. Case 3 – Preoperative sagittal CT image only. Postoperative imaging was unavailable due to follow-up loss.

Arch Plast Surg | DOI 10.1055/a-2867-5301 | © The Author(s).

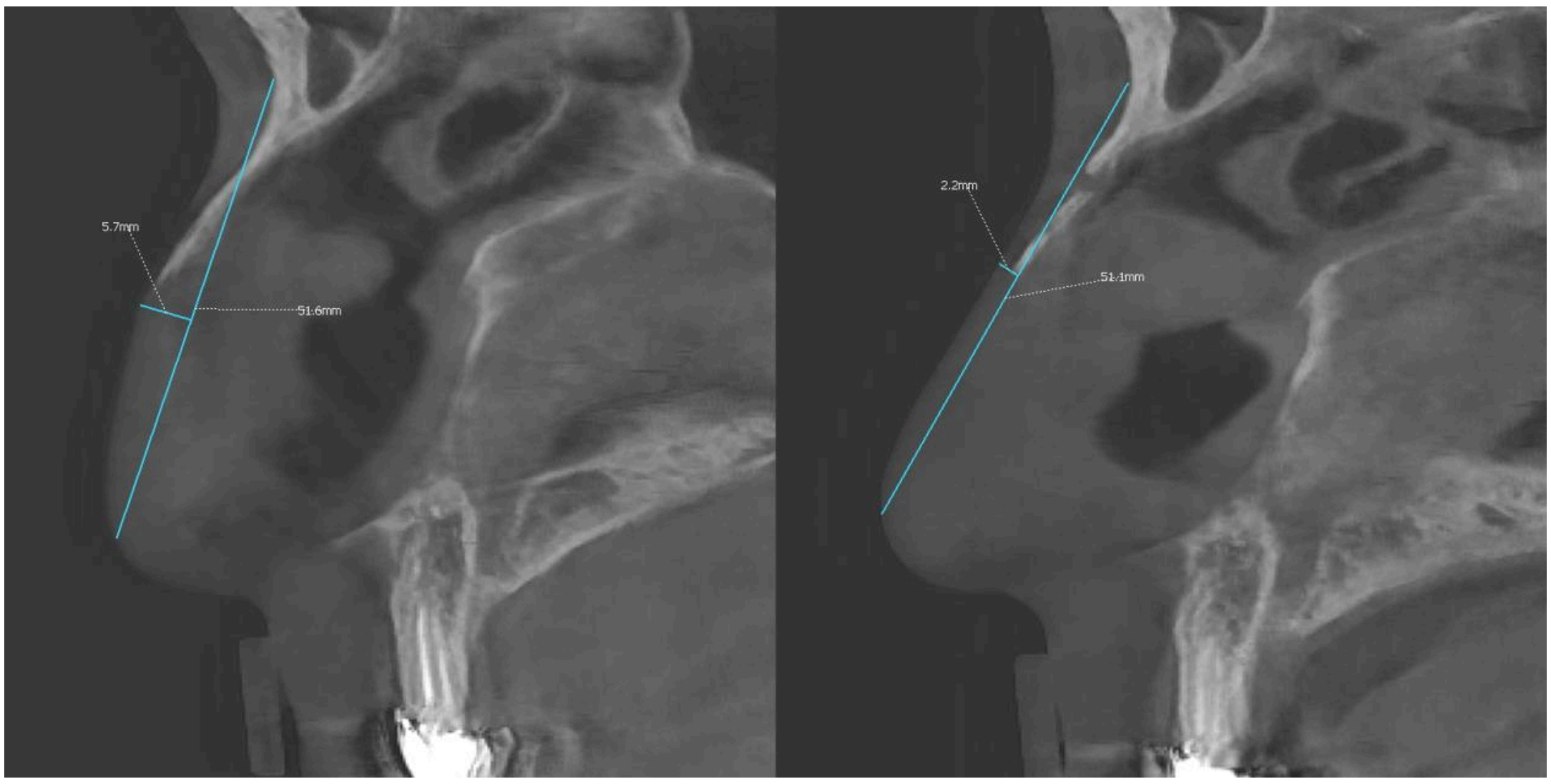

Supplementary Fig. S4. Case 4 – Pre- and postoperative sagittal CT images showing improved dorsal contour (follow-up: 5 months).

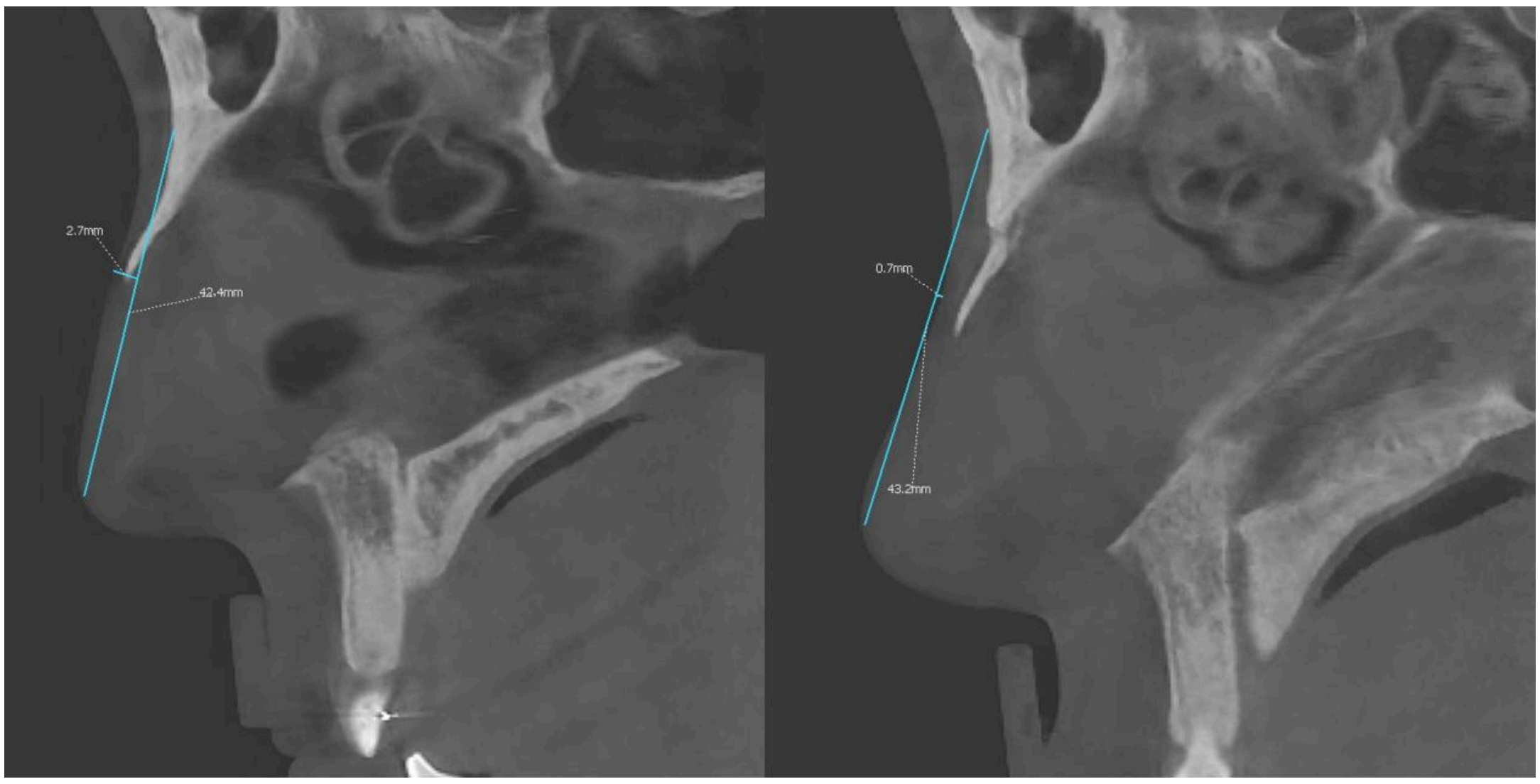

Supplementary Fig. S5. Case 5 – Pre- and postoperative sagittal CT images demonstrating successful dorsal realignment (follow-up: 6 months).

Arch Plast Surg | DOI 10.1055/a-2867-5301 | © The Author(s).
